# Efficient Prediction of Fatigue Damage Analysis of Carbon Fiber Composites Using Multi-Timescale Analysis and Machine Learning

**DOI:** 10.3390/polym16233448

**Published:** 2024-12-09

**Authors:** Satoru Yoshimori, Jun Koyanagi, Ryosuke Matsuzaki

**Affiliations:** 1 Department of Mechanical and Aerospace Engineering, Tokyo University of Science, 2641 Yamazaki, Noda 278-8510, Japan; 7523570@ed.tus.ac.jp; 2 Department of Materials Science and Technology, Tokyo University of Science, 6-3-1 Niijuku, Katsushika-ku, Tokyo 125-8585, Japan; koyanagi@rs.tus.ac.jp

**Keywords:** composites, CFRP, fatigue, numerical analysis, finite element analysis, machine learning, LightGBM, response surface method, entropy

## Abstract

Carbon fiber reinforced plastic (CFRP) possesses numerous advantages, such as a light weight and high strength; however, its complex damage mechanisms make the evaluation of fatigue damage particularly challenging. Therefore, this study proposed and demonstrated an entropy-based damage evaluation model for CFRP that leverages the entropy derived from heat capacity measurements and does not require knowledge of the loading history. This entropy-based fatigue degradation model, though accurate, is computationally intensive and impractical for high-cycle analysis. To address this, we reduce computational cost through multi-timescale analysis, replacing cyclic loading with constant displacement loading. Characteristic variables are optimized using the machine learning model LightGBM and the response surface method (RSM), with LightGBM achieving a 75% lower root mean squared error than RSM by increasing features from 3 to 21. This approach cuts analysis time by over 90% while retaining predictive accuracy, showing that LightGBM outperforms RSM and that multi-timescale analysis effectively reduces computational demands.

## 1. Introduction

Carbon fiber reinforced plastic (CFRP) is used in various fields, including the aircraft [1,2], automotive [3], and medical industries [4,5] because of its advantages in lightweight properties, high strength, and high rigidity. Therefore, it is essential to establish the long-term reliability of CFRP, which is expected to be used in various fields in the future. However, fatigue damage of CFRP in the long-life region has not been sufficiently studied yet. The fracture morphology of CFRP laminates in fatigue is complex [6], unlike that of metallic materials, and the evaluation of damage behavior in terms of fracture mechanics remains insufficient. The fracture of CFRP laminates can be broadly classified into matrix cracks [7,8], which occur at the interface between the matrix and fibers without fiber fracture, transverse cracks [9,10], delamination [11] and fiber fracture [12]. In general, damage propagation in CFRP laminates under fatigue loading depends on the laminate composition. Afterwards, the stress concentration at the tip of the matrix crack causes matrix cracks in adjacent layers, transverse cracks, and delamination, which propagate into the laminate. Finally, fiber fracture leads to the failure of the CFRP laminate [6,13]. Components made from CFRP materials are often subjected to the coupled effects of fatigue load and service environment, including temperature- and humidity-induced corrosion. These adverse environmental effects can accelerate fatigue damage, such as crack formation and propagation [14,15,16]. Thus, it is very difficult to evaluate the fracture mechanics of interacting damage modes. Therefore, resolving these issues is necessary to establish the long-term reliability of CFRP laminates.

Quantitative measurement of damage in CFRP laminates is challenging due to the complexity of failure modes in CFRP fatigue. In addition, it is known that there is a time dependence of damage in the polymeric material, which is the resin portion where the damage begins [17,18]. Therefore, we propose to use the entropy [19,20] generated by the damage of the resin to evaluate the lifetime of the material [21,22].

Entropy can be calculated in two ways. The first is the mechanical method, where mechanical entropy is derived from by dissipated energy. Dissipated energy is the thermal energy generated by the resistance force due to viscosity or friction, which is determined by the stress-strain history, and is a value that can be used to evaluate material damage [23]. The results of fracture simulations performed by Takase et al. [21] confirm that different combined stress states will result in failure at similar values of mechanical entropy. Therefore, mechanical entropy can be used to predict the lifetime of a material under various loading.

Next, the second method is the thermodynamic method. Thermal entropy, using the thermodynamic method is calculated based on the second law of thermodynamics [24]. Thermal entropy can be calculated by measuring heat capacity using differential scanning calorimetry (DSC) [25,26] or the lock-in thermography [27,28,29] even if the loading history is unknown. Molecular dynamics simulation and experimental results by Sakai et al. [30] confirmed that mechanical and thermal entropy have similar values when the same amount of strain is applied [30,31]. Therefore, the damage behavior of CFRP with unknown loading history can be evaluated by comparing the mechanical entropy calculated by cyclic loading simulation with that calculated by measuring the heat capacity.

Koyanagi et al. [32,33,34] developed a fatigue degradation model for CFRP to estimate the remaining life and residual strength of CFRP through an analytical rather than an experimental approach. The proposed model provides a practical approach that determines the strength degradation of CFRP using the entropy damage law; this approach is capable of considering not only single-cycle loading but also various complex loading histories [34]. Indeed, the proposed model enables the estimation of residual strength degradation using only the experimental measurement of entropy without requiring any knowledge of the fatigue history. However, this model has high computational cost and has not been able to analyze the number of cyclic load in accordance with the experiment (example: 100,000 cycles). Therefore, it is necessary to greatly reduce the computational cost of the analytical model.

In this study, we propose reducing computational cost by replacing cyclic loading with constant displacement loading using multi-time scale analysis [35]. In this approach, the characteristic variables are created to be consistent before and after the replacement, but it is difficult to determine because of many variables. Therefore, we propose to use the response surface method (RSM) [36,37,38] and the machine learning model LightGBM [39,40,41] to model the characteristic variables. To reduce the computational cost of the analytical model, the entropy of the resin part of the CFRP was calculated using Abaqus CAE [42] under the same time cyclic loading and constant displacement loading. Next, prediction models of the characteristic variables were created using this data, response surface method and LightGBM. Finally, the prediction accuracy of the response surface method and LightGBM was evaluated by predicting entropy under cyclic loading using the prediction model of the characteristic variables and comparing this value with entropy value under cyclic loading calculated using Abaqus CAE.

## 2. Multi-Timescale Analysis and Prediction Modeling Methods

### 2.1. Multi-Timescale Analysis

**Figure 1:**
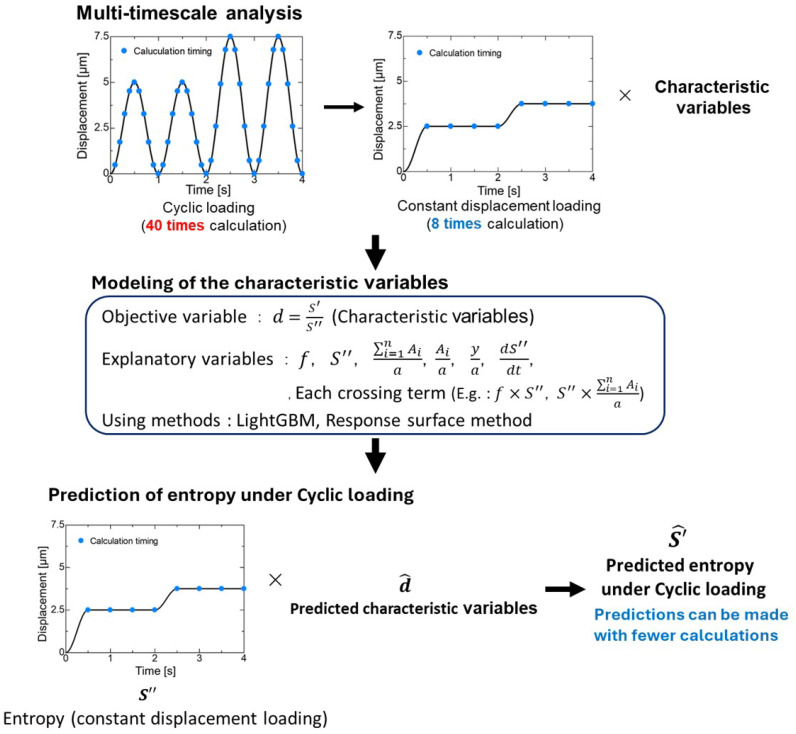
Flow of multi-timescale analysis, modeling of characteristic variables and prediction of entropy under cyclic loading.

Multi-timescale analysis is a method of replacing changes in displacement with constant displacement and characteristic variables on small oscillating timescales, as shown in Figure 1. The characteristic variables are added to the oscillating displacement under constant displacement conditions and modifies material constants to ensure the coherence of the entire system. Using this method, Takamura et al. [35] replaced ultrasonic vibrations with constant stress and characteristic variables, and succeeded in connecting phenomena of the order of $10^{-4}$ with those of the order of $10^{0}$ in their numerical simulations [35]. In this way, the analysis of cyclic loading, which require many calculations with detailed frequency, can be performed with fewer calculations by replacing cyclic loading with constant displacement loading and characteristic variables, as shown Figure 1. As an example, this method can reduce the number of calculation steps from 10 steps per cycle × 100 cycles with a cyclic loading to 1/1000 by replacing it with a single time increment with a constant displacement loading. Given the above, we use multi-timescale analysis to reduce the computational cost of the analytical model. Based on the idea of multi-timescale analysis, we model the characteristic variables and use the model to predict entropy under cyclic loading while reducing computational cost as shown Figure 1. These aspects will be explained in later sections.

### 2.2. LightGBM and Response Surface Method

Next, the modeling of the characteristic variables are described. Determining the characteristic variables in a multi-timescale analysis is challenging because many variables, such as amplitude and frequency, influence the entropy increase. Therefore, we propose using LightGBM and response surface method to create prediction models of the characteristic variables.

**Figure 2:**
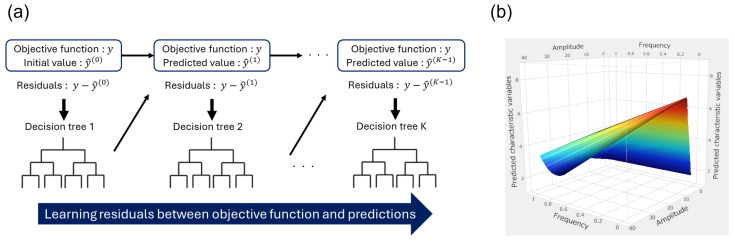
Prediction modeling for the entropy. (**a**) LightGBM. (**b**) Response surface method.

LightGBM [39] is a decision tree-based gradient boosting algorithm and a representative machine learning model. As shown in the Figure 2a, LightGBM models the objective function by repeatedly calculating predictions using decision trees and learning residuals, which are the differences between the predictions and the objective function.

The response surface method [36], on the other hand, generates a response surface as shown in Figure 2b based on response values obtained from experiments and analyses, and creates a regression model as shown in Equation (1). Here, *y* is the response, $x_{i}$ is the explanatory variables, *n* is the number of explanatory variables, $\beta_{0}$, $\beta_{i}$, $\beta_{ii}$, and $\beta_{ij}$ are the regression coefficients. In this study, we used these two methods to predict entropy under cyclic loading and compared their prediction accuracy.

(1) $$\begin{array}{c} y=\;\beta_{0}+\sum_{i=1}^{n}\beta_{i}x_{i}+\sum_{i=1}^{n}\beta_{ii}x_{i}^{2}+\sum_{i<j}^{n}\beta_{ij}x_{i}x_{j} \end{array}$$

## 3. Analysis Conditions

### 3.1. Finite Element Model of CFRP

**Figure 3:**
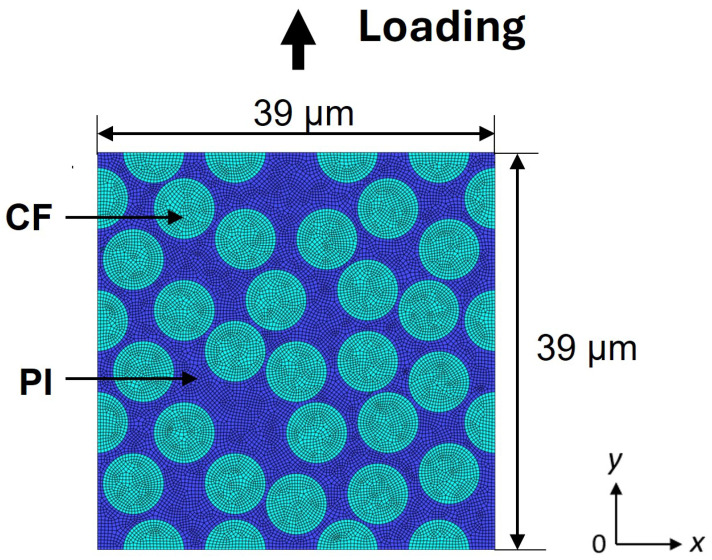
Finite element model of carbon fiber/polyimide composites.

In this study, Abaqus CAE was used to analyze CFRP subjected to cyclic and constant displacement loading. The analytical model used is a finite element model of CFRP as shown in Figure 3, with a 3D unit cell size of 39 μm × 39 μm × 0.3 μm, polyimide (PI) matrix, carbon fiber elastic modulus of 14 GPa, Poisson’s ratio of 0.3, and a volume fraction of 56%. A PI resin matrix was selected for the material model in this study because of its high strength and excellent heat resistance, which make it a promising candidate for composites used in aerospace applications. The element type was set to 8-node brick elements, the number of elements was 18,122, and the number of nodes was 36,718. The PI resin matrix was assumed to be nonlinear viscoelastic-plastic according to a constitutive equation that accounts for entropy damage and was analyzed numerically with the Abaqus/Standard user subroutine UMAT. The finite element model used in this study did not consider interfacial damage between the carbon fibers and PI. The damage estimate obtained using the viscoelastic entropy damage criterion has been studied in prior research and validated through comparisons between experimental and analytical results [22,43]. For details on the constitutive equation incorporating the entropy damage criterion, see [34]. When the element strain reaches 0.25, the element is assumed to have failed and the analysis for that element stops.

**Figure 4:**
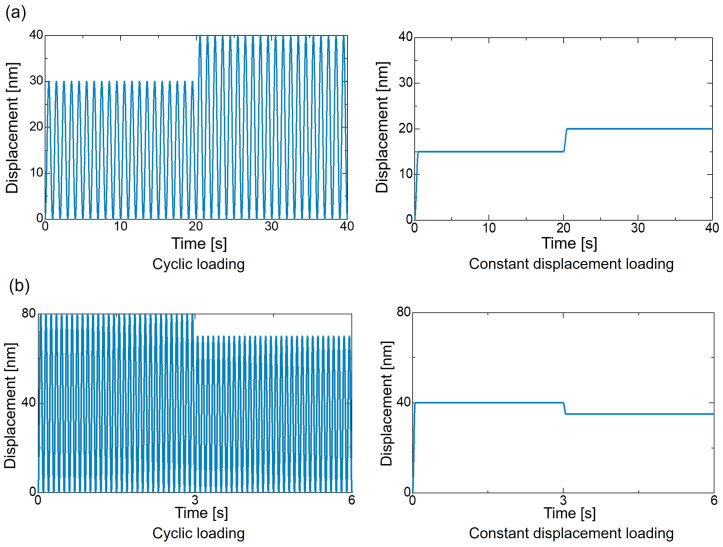
Examples of cyclic and constant displacement loading when the amplitude changed (**a**) from 15 to 20 nm with 20 cycles at 1 Hz each amplitude and (**b**) from 40 nm to 35 nm 30 cycles at 10 Hz each amplitude.

We predicted the entropy of the matrix portion of the CFRP where damage initially occurs. Therefore, the training and test data use the element data of PI that correspond the matrix portion. As shown in Table 1, the cyclic loading was performed at frequencies of 1 Hz and 10 Hz, with the amplitude changing midway while maintaining the same number of cycles at each amplitude. The cyclic loading on the training data was performed for a total of eight conditions, and data for 100 elements of PI were extracted for each condition. In addition, cyclic loading on the test data was performed under a total of four conditions, and data for 30 elements of PI were extracted for each condition. The constant displacement loading conditions for the training and test data were varied in amplitude corresponding to the cyclic loading conditions, and the number of elements extracted for each condition was the same. An example of the change in cyclic and constant displacement loading in Table 1 is shown in Figure 4. Using the training data, a prediction model for the characteristic variables was created. The prediction model was used to predict entropy under cyclic loading under the conditions of the test data. The calculation of the characteristic variables and the creation of the prediction model are described in Section 3.2.

Although data for other frequencies were not included at this stage, we intend to improve the generalizability of the model in future work by incorporating data from additional frequencies to enable expanded predictions under frequencies not present in the training dataset at this time. In addition, the ultimate goal of this study was to enable predictions under realistic cyclic load conditions, such as approximately 100,000 cycles. However, the method for constructing the characteristic variable model and determining the explanatory variables must first be developed and validated. Considering the time required for model development, initial predictions were conducted for a smaller number of cycles than typically observed in actual applications.

### 3.2. Prediction Modeling

In this study, the characteristic variables for each condition and element was defined as in Equation (2).

(2) $$\begin{array}{cc} S_{ij}^{\prime } & =d_{ij}S_{ij}^{\prime \prime } \end{array}$$

Here, $d_{ij}$ is the characteristic variables, $S_{ij}^{\prime }$ is the entropy under cyclic loading, and $S_{ij}^{\prime \prime }$ is the entropy under constant displacement loading. The characteristic variables was calculated using the value of each entropy obtained under the conditions of Section 3.1 and Equation (2). We created prediction models for the characteristic variables by using $\sqrt{d_{ij}}$ as the objective variable and log*f*, $\sqrt{S_{ij}^{\prime \prime }}$, and $\sqrt{\frac{\sum_{i=1}^{n}A_{i}}{a}}$ as the explanatory variables in response surface method, and $d_{ij}$ as the objective variable and *f*, $S_{ij}^{\prime \prime }$, and $\frac{\sum_{i=1}^{n}A_{i}}{a}$ as the explanatory variables in LightGBM. In addition, we also created prediction models in LightGBM with $d_{ij}$ as the objective variable and *f*, $S_{ij}^{\prime \prime }$, and $\frac{\sum_{i=1}^{n}A_{i}}{a}$, $\frac{A_{i}}{a}$, $\frac{y}{a}$, $\frac{dS_{ij}^{\prime \prime }}{dT}$, and each crossing term, for a total of 21 features. Where *f* is the frequency, $A_{i}$ is the amplitude of the cyclic loading, *a* is the length of the analytical model in the tensile direction, and *y* is height from bottom. The response surface method was performed using JMP Pro [44], third-order polynomial regression, and a uniform design of experiments. LightGBM was performed with *K*-fold cross-validation [45] and K=10.

(3) $$\begin{array}{cc} \hat{S_{ij}^{\prime }} & =\hat{d_{ij}}S_{ij}^{\prime \prime } \end{array}$$

The characteristic variables predicted using the response surface method and LightGBM along with Equation (3), were used to calculate the predicted value of entropy under cyclic loading.

## 4. Analysis Results

**Figure 5:**
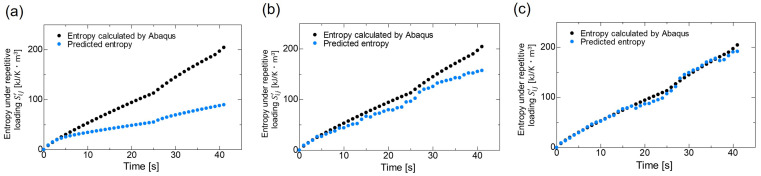
Time variation of entropy under cyclic loading calculated by Abaqus CAE and predicted entropy under cyclic loading calculated by using (**a**) response surface method, (**b**) LightGBM (3 features) and (**c**) LightGBM (21 features) for a single element when the amplitude changed from 12.5 to 17.5 nm with 25 cycles at 1 Hz each amplitude.

As explained in Section 3.2, we predicted entropy under cyclic loading in three ways: using response surface method and LightGBM with three features, and using LightGBM to make predictions with 21 features. In this validation, the prediction accuracy was compared, but the cyclic and constant displacement loading were replaced by the same number of calculations, not verified to reduce computational cost. Figure 5 shows the time variation of entropy under cyclic loading calculated by Abaqus CAE and predicted entropy under cyclic loading calculated by using each of the three methods for a single element given the same conditions. In this study, we predicted entropy under cyclic loading per one cycle as shown in Figure 5 for each condition and each element.

(4) $$\begin{array}{cc} RMSE & =\sqrt{\frac{1}{n}\sum_{i=1}^{n}{\left(y_{i}-\hat{y}\right)}^{2}} \end{array}$$

**Figure 6:**
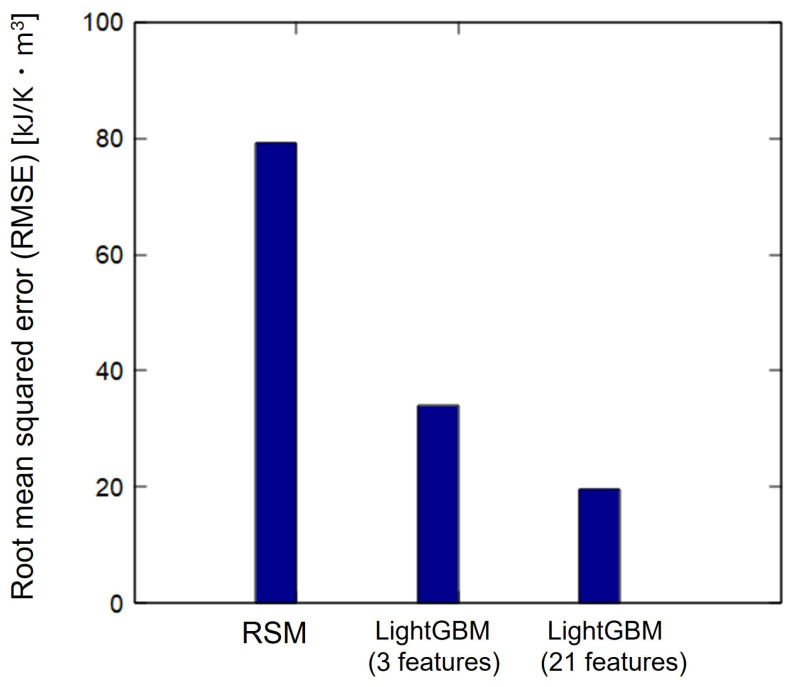
RMSE when response surface method, LightGBM (3 features) and LightGBM (21 features) are performed.

In this study, root mean squared error (RMSE) was used to quantitatively evaluate the prediction accuracy for each method. Here, $y_{i}$ is the actual value of entropy under cyclic loading, $\hat{y_{i}}$ is the predicted value of entropy under cyclic loading, and *n* is the number of data. RMSE is the average of squared residuals, as shown in Equation (4), and is more strongly affected by outliers. Therefore, RMSE values was used in this study to evaluate prediction accuracy by also considering outlier. Figure 6 shows the RMSE for each of the three methods. RMSE were smaller for LightGBM than for the response surface method. Therefore, LightGBM has better prediction accuracy than the response surface method. This is because LightGBM, a decision tree-based method, effectively captures nonlinear phenomena, making it more suitable for entropy prediction than simple polynomial regression used in response surface methods. Additionally, by increasing the number of explanatory variables in the LightGBM from 3 to 21, the RMSE decreased significantly. RMSE decreased by approximately 75% compared to RSM and by approximately 42% from LightGBM (3 features). The validation conducted in this study indicated that the LightGBM model (21 features) exhibited the highest accuracy with an RMSE of 22.3 $\mathrm{kJ}/(K\;\cdot \;m^{3})$. Given that the fracture entropy ranged from approximately 220 to 300 $\mathrm{kJ}/(K\;\cdot \;m^{3})$, this error corresponds to approximately 10%. The comparison of the model-predicted entropy values with those obtained through heat capacity measurements confirmed that the proposed model can be applied to predict entropy under realistic cyclic load conditions, enabling the evaluation of damage to materials with unknown load histories.

**Figure 7:**
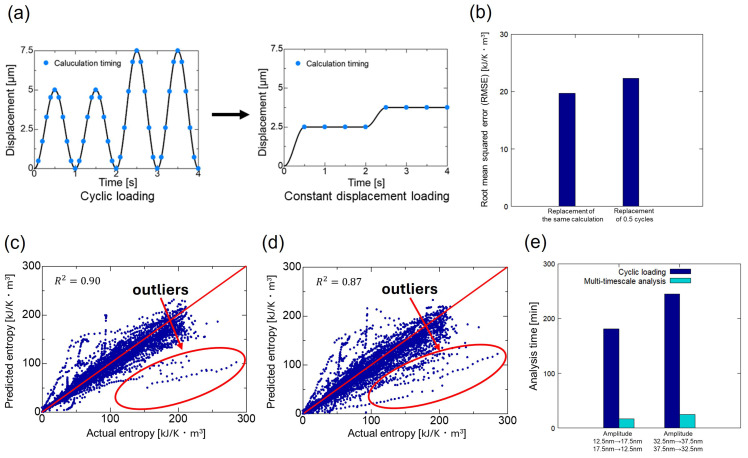
Results of computational cost reduction measures. (**a**) Replacing cyclic loading with constant displacement loading. (**b**) RMSE of predicted entropy. Actual and predicted entropies when replaced by (**c**) the same number of calculations and (**d**) fewer calculations. (**e**) Analysis time.

Next, we verified the reduction in computational cost using LightGBM (21 features), which had the highest prediction accuracy. As shown in Figure 7a, the cyclic loading, which calculates approximately 10 times per cycle, was replaced by constant displacement loading, which calculates once every 0.5 cycles, along with the characteristic variables. First, we examined the change in prediction accuracy as a result of replacing the data with a different number of calculations. Figure 7b shows the RMSE of cyclic loading and constant displacement loading replaced by the same number of calculations and cyclic loading replaced by constant displacement loading calculated once every 0.5 cycles. RMSE was larger for the case where by constant displacement loading was calculated once every 0.5 cycles, but the difference was not significant, and no major loss of prediction accuracy was observed. Also, we examined the relationship between the actual entropy under cyclic loading calculated using Abaqus CAE and the predicted entropy under cyclic loading. Figure 7c show the relationship between entropy under cyclic loading calculated by Abaqus CAE and predicted entropy under cyclic loading when replacing cyclic loading and constant displacement loading with the same number of calculations. Figure 7d shows the relationship between entropy under cyclic loading calculated by Abaqus CAE and predicted entropy under cyclic loading when replacing cyclic loading with constant displacement loading that only performs one calculation every 0.5 cycles. The coefficient of determination ($R^{2}$) shown in Equation (5) was calculated to quantitatively evaluate the prediction accuracy.

(5) $$\begin{array}{cc} R^{2} & =1-\frac{\sum_{i=1}^{n}{\left(y_{i}-\hat{y_{i}}\right)}^{2}}{\sum_{i=1}^{n}{\left(y_{i}-\bar{y_{i}}\right)}^{2}} \end{array}$$

Here, $y_{i}$ is the actual value of entropy under cyclic loading, $\hat{y_{i}}$ is the predicted value of entropy under cyclic loading, $\bar{y_{i}}$ is the average value of entropy under cyclic loading, and *n* is the number of data. $R^{2}$ takes values between 0 and 1, and the closer to 1, the better the prediction accuracy. $R^{2}$ is the value that explains the variability of the model compared to RMSE. $R^{2}$ decreased from 0.90 to 0.87, but not significantly. Comparing the red points in Figure 7c,d, there were more outliers in case (d), indicating larger deviations from the unit slope curve when replacing cyclic loading with constant displacement loading that only performs one calculation every 0.5 cycles. Therefore, reducing outliers to further improve prediction accuracy is an issue for the future. Regarding the accuracy of the machine learning model proposed in this study, similar work by Amin et al. [46] predicted the interfacial bond strength of FRP composites using LightGBM and XGBoost; the former achieved an $R^{2}$ value of 0.865 against the test data. In this study, the computational cost-optimized LightGBM model achieved an $R^{2}$ value of 0.87 against the test data, demonstrating comparable accuracy.

Next, we examined the extent to which computational costs were reduced. Figure 7e shows the analysis time for each amplitude change when calculating entropy with cyclic loading in Abaqus CAE and when predicting entropy with cyclic loading at constant displacement loading calculated once every 0.5 cycles using multi-timescale analysis. The analysis time was significantly reduced when calculated using constant displacement loading in the multi-timescale analysis compared to the actual repeated loading analysis using Abaqus CAE. Specifically, when the amplitude was changed from 12.5 to 17.5 nm and from 17.5 to 12.5 nm, the analysis time was reduced to approximately one-tenth. When the amplitude was changed from 32.5 to 37.5 nm and from 37.5 to 32.5 nm, the analysis time was reduced to approximately one-ninth.

## 5. Conclusions

The optimal method to predict entropy under cyclic loading was verified to reduce the high computational cost associated with fatigue degradation modeling of CFRP by using multi-timescale analysis and prediction model of characteristic variables. As a result, LightGBM had smaller values for RMSE than RSM, and better prediction accuracy than RSM. This study used a large dataset to develop the evaluated models and found that LightGBM, which employs decision trees and is better suited to capturing nonlinear phenomena, was more appropriate for entropy prediction than RSM that perform simple polynomial regression. Also, by increasing the number of explanatory variables in the LightGBM from 3 to 21, RMSE decreased by approximately 75% from RSM and by approximately 42% from LightGBM (3 features). As a result of the verification of the computational cost reductions, the analysis time was reduced to less than one-ninth by replacing cyclic loading, which is calculated approximately 10 times per cycle, with constant displacement loading, which is calculated only once every 0.5 cycles. Additionally, no significant decrease in prediction accuracy was observed from RMSE and $R^{2}$. Based on the results obtained in this study, we intend to predict the entropy values of test specimens subjected to a larger number of cyclic loads that cannot be analyzed otherwise owing to computational cost constraints. This will enable further validation of the proposed method for CFRP damage prediction.

## Figures and Tables

**Table 1:** Cyclic loading conditions for training and test data.

**Learning Data**	
**Frequency [Hz]**	**Amplitude Change [nm]**
1	10 (30 cycles) → 15 (30 cycles)
1	15 (30 cycles) → 10 (30 cycles)
1	15 (20 cycles) → 20 (20 cycles)
1	20 (20 cycles) → 15 (20 cycles)
10	30 (40 cycles) → 35 (40 cycles)
10	35 (40 cycles) → 30 (40 cycles)
10	35 (30 cycles) → 40 (30 cycles)
10	40 (30 cycles)→ 35 (30 cycles)
**Test Data**	
**Frequency [Hz]**	**Amplitude Change [nm]**
1	12.5 (25 cycles) → 17.5 (25 cycles)
1	17.5 (25 cycles) → 12.5 (25 cycles)
10	32.5 (35 cycles) → 37.5 (35 cycles)
10	37.5 (35 cycles) → 32.5 (35 cycles)

## Data Availability

The raw data supporting the conclusions of this article will be made available by the authors on request.
